# Lectures on the Structure and Physiology of the Male Urinary Organs

**Published:** 1822-09-01

**Authors:** 


*Lectures on the Structure and Physiology of the Male Urinary and Genital Organs of the Human Body, and on the Nature and Treatment of their Diseases; delivered before the Royal College of Surgeons in London, in the Summer of the Year* 1821. By James Wilson, F.R.S. Professor of Anatomy and Surgery to the College; Lecturer on Anatomy and Surgery at the Hunterian School in Great Windmill Street; and one of the Vice-Presidents of the Medico-Chirurgical Society of London. One Vol. 8vo. pp. 438, three Plates. London, September 1821.

This is the third and final volume of Mr. Wilson's Lectures before the College of Surgeons. It is, also, not the least valuable and interesting of the three. We are happy to find, that the opinion which we expressed of its two predecessors, has been amply confirmed by the profession at large—their reception by the public having been most favourable. The present work is dedicated to Henry Cline, Esq. one of the Patriarchs of Surgery, whose extensive knowledge adds lustre to the profession, and is only surpassed by his liberality of sentiment and integrity of principle. Happy would it be for the faculty, if the junior members would look to the examples of those distinguished individuals who are at the head of the profession, and imitate their conduct to each other, and to the public at large! We hope and trust, that there is a growing spirit of urbanity and magnanimity diffusing its genial influence among the ramifications of medical society, and which will one day harmonize its members, to an extent far beyond what is contemplated by those, who keep their eye fixed on the dark side of human nature, and neither hope nor strive to brighten the prospect.

It is evident, that lectures, delivered before such a body as the Royal College of Surgeons, must be divested of opinions founded in ignorance, and secured from the pernicious effects resulting from empirical practice, however artfully and speciously disguised. They come, therefore, in a shape and garb, (independently of the character, experience, and industry of the author,) which render their reception doubly welcome, since it has but too often happened, that these subjects have been presented to the public and to the profession, enveloped in the mystery with which ignorance, knavery, or quackery, chose to clothe them.

The work consists of fifteen lectures, and embraces an amazing range of important matters, which it would be impossible to even enumerate, much less analyze, in an article of this kind. We must, therefore, be very concise, as well as desultory in our extracts, and notices of the contents of this volume.

In the *first* lecture, after some introductory observations, Mr. Wilson presents a neat analysis of what has been written on the subject of urine itself, by Wollaston, Bostock, Brande, Marcet, Prout, Berzelius, &c. He then takes up the anatomy of the kidney, ureters, and bladder, which occupy the first and second lectures. In the third lecture, Mr. Wilson enters on the anatomy and physiology of the genital organs, viz. the scrotum, spermatic chord, and testicles. The fourth chapter treats on secretion generally—on the structure of the testicle—on its first formation; and on its descent to the scrotum. The fifth lecture is on the vesiculæ seminales, prostate gland, and Cowper's glands. The sixth lecture delineates the structure and functions of the various parts composing the membrum virile, and here ends the strictly anatomical and physiological part of the course, into which we could not, for obvious reasons, enter in this article. We need hardly observe, that Mr. Wilson's anatomy is rigidly correct, while his physiology is that which is founded on the best authenticated facts hitherto discovered, and the opinions of the best physiologists who have written on these subjects.

In the seventh lecture, Mr. Wilson commences the pathology of the genito-urinary organs, and first of all, offers some general remarks on calculous concretions. So much has been introduced into this Journal, of late, on the subject of urinary calculi, that we shall pass over this part of Mr Wilson's work, remarking merely, that the best information is drawn from various and respectable sources, and condensed into a narrow compass. We shall extract the following case, shewing how a calculus, and that of the mulberry species too, may lie for many years in the bladder without causing much inconvenience—at least in the organs immediately concerned.

"Several years ago I examined, along with Mr. Cruikshank, the body of a gentleman who died of a dysentery at the advanced age of eighty-one. For some years before his death he was a constant attendant on the lectures delivered by Dr. Baillie and Mr. Cruikshank in Windmill Street, when I was the demonstrator of anatomy in that school. I frequently, at that time, dissected bodies for him; and, before his death, received a paper from him with a detailed account of all the symptoms of disease which he could remember as having occurred to him during the last forty years of his life, with a view of having the parts affected by them examined by Mr. Cruikshank and myself after his death, he having inserted a clause in his will to that purport. I mention this, particularly as among the various parts of the body he had named, neither the kidnies nor bladder were alluded to; and as he had long been professionally attended by Mr. Cruikshank and myself, had any difficulty of passing his urine occurred, it would not have been concealed from us. Notwithstanding this, a very dark coloured oxalate of lime or mulberry calculus was found in his bladder, the bladder itself exhibiting no particular diseased appearances. The projections from this calculus were rather long spicula with blunted points than tubercles; and probably from their shape, preventing at any time the whole surface of the calculus from touching the sides of the bladder, allowed the urine to pass into the urethra, so that no obstruction to its flow ever took place. What could have prevented such calculus from irritating and bringing on the other symptoms of stone I know not; but I have met with other instances where the oxalate of lime calculus has produced but little comparative irritation, although, I believe, it is a generally received opinion that it produces the greatest." 236.

A recent case of this kind occurred in the practice of Dr. Paris and Mr. Chilver. The patient was walking in St. James's Park on the 6th of April, 1820, and feeling fatigued, went to sit down on one of the benches, which, from its unexpected lowness, gave him a considerable shock, that probably dislodged the calculus from the sac in which it had reposed. He was immediately seized with painful dysury, which continued till his death, six days after the accident. In this patient, the existence of a calculus was never suspected, till Mr. Wilson passed the catheter after the accident happened, and struck against the stone. A calculus of the mulberry kind, weighing 631 grains, was taken from the bladder after death. The interior surface of the bladder was found much inflamed, and a sac was discovered in which the stone had lodged.

The following is the pathological state of the bladder, after the long-continued irritation of a calculus.

"The constant irritation of the coats of the bladder produces a considerable thickening in their substance, but principally in the muscular coat, the packets of its fibres becoming very large, and incapable of that dilatation which they formerly possessed; their irratability however increases, so that they are excited to contract upon a few drops of urine; and thus, by pressing the stone against a part already too sensible to pain, an almost constant state of suffering is kept up. The bladder in time becomes more diseased, the inner coat more constantly inflamed and sometimes ulcerated, all the unfavourable constitutional symptoms increase; and unless an operation is performed which removes the stone, the patient's sufferings are only ended by death." 239.

Mr. Wilson here introduces a brief view of the treatment of calculous complaints, chiefly compiled from the writings of Brande, Marcet, Prout, and the best modern authors. In respect to the high operation, Mr. Wilson does not give a decided opinion. He seems to think that the high and the lateral operations have been attended with nearly equal success.

We may here be permitted to make a short digressive allusion to the subject of the recto-vesical operation of Vacca, described in our last number. Since that paper was printed, we have been informed by Sir Astley Cooper, that the operation, as detailed by Vacca, was performed by him at Guy's Hospital, twelve years ago, and the man died. It was also performed at the London Hospital, by Mr. Headington, and the wound never closed afterwards. We have also been informed by a very eminent surgeon of this metropolis that, in the lateral operation, he, by accident, during a jerk of the patient, carried the external incision into the rectum, and the consequence was, a fistulous opening into the urethra at that part, which continued, in spite of every means which could be employed to close it. We throw out these hints, not to discourage a trial of the operation, but to put surgeons on their guard. Perhaps, the precaution which Vacca used, of touching that part of the wound which corresponds to the incision of the rectum, and that portion which remains in the perineum, with lunar caustic immediately the danger of inflammation is over, and suppuration is established, may have greatly tended to obviate urinary fistulæ, as sequelæ of this operation.

The tenth lecture is "on excess of albumen and urea in the urine," and on some affections of the kidnies. Mr. Wilson justly remarks, that in *studying* the nature of diseases we cannot observe those arbitrary and artificial distinctions between physic and surgery, which common usage and mutual consent have set up in *practice*, in this and some other countries. Local and constitutional diseases must be studied by both physician and surgeon, as well as the general practitioner.

On the disposition to pass albuminous urine, Mr. Wilson makes some observations; but acknowledges how little we know of the means of counteracting it. This inordinate separation of albumen from the blood often occurs in dropsical patients, and our readers know that Dr. Blackall considers the phenomenon as indicative of venesection. But the Dublin Hospital physicians have long since shown the total uncertainty of this indication, and we believe it is now generally disregarded by the most experienced physicians.

"We know," says Mr. Wilson, "that in local inflammations the coagulable lymph is separated from the blood, and it may be also separated in the kidneys, if their secreting action is too strongly excited, and then bleeding might do good; but before this plan is adopted, it should be ascertained whether the dropsy is the cause of the separation of albumen, or the effect; if it proves to be the cause, by bleeding we should increase the disease. I have seen instances, in which the muriated tincture of steel proved very useful in lessening, and I believe in removing, this complaint." 265.

Excess of urea is not unfrequently met with, especially in children, and in people depositing the phosphates. In these cases the urine is generally pale—sometimes high-coloured, like porter and water. When recently voided it reddens litmus paper, and is, for the most part, free from sediment. Nitric acid produces speedy crystallization, and then an abundance of urea is discovered. Dr. Prout thinks that excess of urea has been confounded with diabetes insipidus, though greatly differing from that disease.

"Where urea is in excess, there is usually a frequent and almost irresistible desire of voiding the urine: this does not arise from fulness of the bladder: for, in general, a small quantity is voided at any one time; but from the frequency, the total quantity voided in a given time is greater than natural. This quantity is augmented in cold weather, and is also increased by all causes producing mental agitation. There is often a sense of weight or dull pain in the back, and an occasional irritation about the neck of the bladder, which sometimes extends along the urethra. The pulse however is not affected, and the tongue is clean: there is no remarkable thirst, nor is there any craving for food, nor are the functions of the stomach and bowels much deranged." 266.

In Dr. Prout's experience a majority of the cases of this kind, were persons who early in life had been addicted to habits which weaken the genital organs. He thinks that the train of symptoms, if suffered to proceed, may sometimes terminate in diabetes, or in a deposition of the phosphates. Stimulating remedies, such as the copaiba, have been found to increase the complaint. Sedatives, especially opium and hyoscyamus, combined with aperients, are most efficient in suspending, if not removing the complaint. On diabetes, Mr. W. makes a few remarks. He has inspected three bodies who died of this distressing disease, and in none of them were the kidneys enlarged.

"In two of these I found that vascular appearance which Dr. Baillie has described, where the superficial veins were much fuller than usual of blood, forming upon the surface of the kidney a most beautiful net-work of vessels. In one of these cases the renal capsula was enlarged, and contained a fluid in a circumscribed cavity, which in colour was like the bile when mixed with water; and in both cases the liver was sound. The spleen was sound in one of these, but tuberculated in the other. In the third case, the liver was tuberculated, and much shrunk in size; the lungs also were filled with scrofulous tubercles." 269.

It is needless to say that this is a most indomitable disease. In fact, we consider it generally as a breaking up of the constitution, or of some vital organ, and therefore it is one of the numerous outlets by which Nature removes us from the moral, and mingles us with the physical world.

The various organic diseases of the kidney, which Mr. Wilson has noticed, we must pass over. Our attention, however, was strongly arrested by a melancholy case of ignorance and temerity detailed at page 288—a case not very likely to happen in these days, when medical and chirurgical knowledge is so widely diffused. One would hardly suppose that suppression and retention of urine could be confounded together—yet Mr. W. has known an instance of the mistake involving the life of the patient.

A musician of some celebrity, after some intemperance, became alarmed at not having made water for some hours. The surgeon and apothecary (now dead) tried the usual means employed for retention of urine without effect. He then introduced a catheter into the bladder (as he thought) with much difficulty. No urine flowed, but some fæculent matter was found adherent to the sides of the instrument.

Immediately after this the abdomen swelled, and symptoms of peritoneal inflammation came on. Dr. Pearson and Mr. Wilson were called in, and found the patient *in articulo mortis*. There was no tumour above the pubis, nor was the bladder found distended, when examined per rectum. Mr. Wilson passed the catheter into the bladder with ease, but no urine followed. The patient died; and, on dissection, a hole was found in the coats of the bladder, on the back part of its cavity, between the entrance of the ureters, corresponding with the size of the catheter. The hole communicated with the cavity of the abdomen, as it was above the reflection of the peritoneum from the rectum to the bladder. The peritoneum round it was much inflamed, and partially adhering to the rectum. Another hole, exactly corresponding to the first, was found on the fore part of the rectum, entering the cavity of the gut. Both holes seemed to have been made by the same instrument. The case was evidently a suppression and not a retention of urine originally.

No general rules can be laid down for treating suppression of urine. In those cases dependant on inflammation of the kidney, bleeding from the arm and from the loins will sometimes restore the secreting power of the kidney. Aperient medicines will also be necessary. In general, however, this dangerous symptom is a prelude to death from other diseases.

On retention of urine, Mr. Wilson makes many good remarks, but none of them containing any novelty.*

The 12th lecture is on diseases of the bladder and prostate gland, of which we cannot offer any analysis, the matter not being adapted for that process. The 13th lecture is in continuation, and on strictures of the urethra, for which we must refer to the work itself. The 14th lecture continues the subject of stricture, embraces fistula in perinæo, and takes in the operation of puncturing the bladder.

Respecting caustic bougies, we apprehend that not all the preparations placed in the college museum, by Sir E. Home,

---

* We knew an old and experienced army surgeon, who assured us that, in his whole life, he never saw an instance where retention of urine, if left to itself, or treated by the common means, failed to do well, without any recourse to the knife. He affirmed that puncturing the bladder, *never* was necessary in retentions of urine. Although we cannot agree with him so far as this, yet we believe that cutting into the bladder is sometimes performed prematurely, or rather unnecessarily. The observations of our old military friend were recalled to our memory lately, by hearing a discussion on this subject in a medical society of the metropolis, where a similar doctrine was maintained by an individual present, to the astonishment of many of the surgeons around him.—*Rev.*

will induce the surgeons of the present day to embrace again the baronet's mode of treatment. Were the *per contra* preparations, which now sleep in the silent tomb, arranged in opposition to those in the college, what a fearful balance would there be against the caustic! But we need not arraign the fallen. The armed bougie is no more.

The last or 15th lecture is on diseases of the testicle—and impotence from bodily and from mental causes. On these subjects Mr. Wilson delivers, as usual, solid information, but without any pretensions to novelty. This work, like those that preceded it, is a good text book for the young, and a useful remembrancer for the old practitioner.

P. S. The foregoing short notice of Mr. Wilson's work had been prepared for our last number, but we had not room for its insertion. We have now the melancholy task of recording the death of a man, from whom we imbibed our first rudiments of anatomy, and whom we never ceased to respect as a master and esteem as a friend. His able colleague, Mr. Charles Bell, in a pathetic address to his pupils, as published in the Medical Repository for December last, informs us that Mr. Wilson was a native of Ayrshire, in Scotland, and came to town when very young, under the protection of the late Mr. Cruikshanks, who was, first, librarian, then assistant to Dr. William Hunter. At the death of Mr. Cruikshanks, Mr. Wilson became assistant lecturer with Dr. Baillie, who finally, and in a most liberal manner, resigned the professorship to Mr. Wilson. This gentleman carried on the anatomical lectures with great credit to himself and advantage to the public for nine years, viz. till 1812, when Mr. Charles Bell purchased Mr. Wilson's interest in the school, since which time these gentlemen conducted the anatomical tuition with harmony among themselves and satisfaction to the numerous pupils of this distinguished school. We shall quote the following passage from Mr. Bell's address, for the accuracy of which we ourselves can vouch from 25 years' personal knowledge of the deceased.

"In speaking of my late colleague's character for science and a knowledge of anatomy, it is a consolation to me, that I have only to repeat what I delivered before you while he was yet with us, which he heard through some of you, and which gratified him: I say, it is a satisfaction to be able to use the same words of praise while our friend lives, as after his death. It proves that we do not see through the magnifying influence of a recent loss, nor allow ourselves to be affected by vain regrets. He was, what few are, early and regularly taught anatomy,—had the best teachers in his youth, and a correspondence with the first men while he lived. As I said to you on a former occasion, there descended through him, by a regular succession of pupillage, those views of the anatomy, that accuracy and minuteness of knowledge, and those methods of teaching, which had characterized the Hunters, and those who immediately succeeded them,—and to whom this country owes what credit it has obtained for a knowledge of the human structure.

"You can testify, Gentlemen, the precision and minuteness of his demonstrations, and the rigid manner in which he performed his duty to *you*, exacting attention to the last minute of that time which ought to be given to the lecture, and never permitting his endeavours to relax when your interests were concerned.

"His discoveries did not embrace any great system: but, what was of more advantage, they extended to every department of the demonstrations of the anatomy: and this is particularly evinced in the collection of anatomical preparations which he made, and which is still before you.

"He was Professor of Anatomy to the College of Surgeons; and there, where the College have drawn together the most eminent to grace their Institution, he was distinguished by the propriety of his manner, suiting his own character and the place; but not in manner only was he distinguished as a Professor, but in method and matter also.

"My colleague was a man of great good nature, in the best sense of the word. He had no envy or repining at the success of others; and many whom he had taught, and whom he had assisted in early life, knew better than he did, by what means fortune was to be attained.

"He had his full share of the harrassments of a professional life. It was in the country, and retired with his family, in his little, simple, rural cottage—that his friends, and especially those younger friends, some of whom I address, will most love to remember him. There his cheerfulness and hospitality, with a manner kind, attentive, and without ostentation, exhibited the goodness of his heart; while his willingness to promote and to partake of every youthful gratification evinced the simplicity of it,

"No man had warmer friends; and this is the best praise a man can have—warm and steady friends through life:—the names of Dr. Baillie, Mr. Cline, and Mr. Abernethy, among these, are sufficient eulogy of my departed colleague.

"He has left a son, who has been remarkable in every situation from early youth, where scholarship, taste, and correct conduct could give distinction: just entering life, he has sustained the heaviest loss any man can suffer; but fortunate in this, that his family can look forward with confidence to him as their comfort and support.

"The last time you saw Mr. Wilson in this place, he complained to me of his head, and pointed to a particular spot; the frequent return of this pain he attributed to the severe shock he had received on his carriage breaking down some time ago.

"On Thursday morning he had complained of being unwell—he felt his hand unsteady, but he breakfasted, shaved, and had sat down to write a letter to a patient, when he felt himself taken exceeding unwell; he rose, rung the bell, and sent for his son, saying, he was very ill, but desiring that Mrs. Wilson and his daughters should not be alarmed.

"Mr. Wilson called his house pupils to his assistance, and finding his illness still increasing, he sent for Dr. Baillie. In the mean time he was bled and cupped. On Dr. Baillie's arrival he was still capable of speaking, and laid his hand on his left breast, as the seat of his uneasiness. Dr. Baillie prescribed for him—a blister was put on the nape of his neck, and sinapisms to his feet.

"In an hour Dr. Baillie returned, and other physicians saw him; but he was sinking, and died at half-past twelve.

"On examination of the body, the heart and vessels were found unusually empty of blood, and a serous effusion had taken place on all the surfaces of the brain: with the exception of some ossifications of the valves of the heart, all the viscera appeared natural." 514. *Med. Repos. Dec.* 1821.

Thus has been suddenly called away from us, in the zenith of his faculties, one of the best anatomists and ablest teachers of the present enlightened æra.

Abstulit clarum cita mors Achillem!

But what availeth grief for the dead? It is a wise and happy dispensation of the God of Nature that the daily sight of death induces not despair in the living. On the contrary, every one of us secretly aspirates, to the latest period of our existence, the following sentence of the poet:—

Et mihi forsan, tibi quod negârit porriget hora.—Hor.

